# The Effectiveness of Mobile Health (mHealth) in Reducing Alcohol Consumption Among Adults in Developed Countries: A Systematic Review

**DOI:** 10.7759/cureus.90110

**Published:** 2025-08-14

**Authors:** Khairina Khairuddin, Khong Wee Lee

**Affiliations:** 1 Public Health, Birmingham City University, Birmingham, GBR; 2 Trauma and Orthopaedics, Ysbyty Gwynedd, Bangor, GBR

**Keywords:** alcohol use disorder, behaviour change techniques, digital interventions, mhealth, mobile health, public health interventions, public health strategy

## Abstract

Alcohol use disorder (AUD) remains a major public health issue, contributing to significant morbidity, disability, and premature mortality. With the proliferation of mobile health (mHealth) technologies, smartphone applications have emerged as innovative platforms for delivering alcohol reduction interventions. Despite growing interest, existing evidence regarding their effectiveness is inconsistent and scattered, highlighting the need for a synthesis of the literature. A systematic review was conducted using multiple electronic databases, including the Cochrane Library, PubMed, and PsycINFO, with coverage extending through December 2024. Eligible studies included randomised controlled trials (RCTs) that assessed the impact of mobile application-based interventions on alcohol consumption. Screening followed predefined inclusion and exclusion criteria. Data extraction and critical appraisal were performed using the Cochrane risk of bias 2 (RoB 2) tool, the gold-standard framework for evaluating methodological quality in randomised studies. From an initial pool of 49 studies, eleven met the inclusion criteria and underwent full appraisal, with ten randomised controlled trials ultimately included in the synthesis, encompassing a total of 11,269 participants. One study was excluded due to a high risk of bias identified across multiple domains. The selected interventions were grounded in diverse behaviour change theories (BCTs), including brief intervention (BI), cognitive behavioural therapy (CBT), cue exposure therapy (CET), social cognitive theory, social norms theory, protective behavioural strategies (PBS), the information-motivation-behavioural skills (IMB) model, motivational interviewing (MI), and the capability, opportunity, motivation, and behaviour (COM-B) framework. Narrative synthesis revealed that individuals engaging with mHealth interventions tended to consume less alcohol compared to those in control groups or receiving minimal intervention. However, due to heterogeneity in theory application and outcome measures, further evidence is needed to identify which BCTs yield the most consistent and effective reductions. This review supports the potential of mHealth applications in facilitating reductions in alcohol consumption among adults in high-income settings. Nonetheless, the diversity of study designs, theoretical models, and outcome metrics underscores the need for greater methodological standardisation and extended follow-up periods. Future research should focus on refining intervention components, enhancing reporting transparency, and evaluating long-term effectiveness to better inform policy and practice in digital public health.

## Introduction and background

Alcohol use among adults in developed countries remains a significant public health concern, shaped by sociocultural norms, stress, and economic access [1]. The World Health Organisation (WHO) attributes 2.6 million annual deaths and 133 million disability-adjusted life years (DALYs) to alcohol consumption, with over half occurring in those under 60 [2]. In nations such as the UK and Switzerland, alcohol use is reported by 81% and 83% of adults, respectively [3,4].

Excessive drinking is linked to liver disease, cardiovascular issues, and mental health disorders. Its bidirectional relationship with psychiatric conditions reinforces a cycle of misuse, compounded by domestic violence and antisocial behaviour [5], which may negatively impact child development [6]. The broader societal cost amounts to an estimated 2.6% of GDP [1], underscoring the need for effective interventions.

Mobile health (mHealth) tools offer scalable, personalised support to reduce alcohol use. Defined by Ryu through the WHO’s eHealth Observatory [7], mHealth apps integrate features such as self-monitoring, goal-setting, and feedback to promote behaviour change. With over 350,000 health apps globally [8], these platforms show promise in addressing barriers like stigma and poor treatment uptake [9,10].

Despite global action plans, per-capita alcohol consumption has increased and is projected to reach 7.6 litres by 2030 [1]. Cultural norms, permissive attitudes, workplace stress, and marketing efforts contribute to hazardous alcohol consumption (HAC). The Theory of Normative Social Behaviour suggests that group identity and peer similarity amplify these risks [11].

Although many mHealth apps exist, few are evidence-based, and prior reviews are often outdated. Sawares et al. [12] and Hingson et al. [13] advocate for early, discreet interventions, where mHealth offers a viable supplement to conventional strategies. This systematic review evaluates the effectiveness of mHealth applications in reducing alcohol consumption among adults in developed countries, identifying the most impactful behavioural models and informing future digital health strategies.

## Review

Materials and methods

This systematic review was registered in the International Prospective Register of Systematic Reviews (PROSPERO) database (CRD420251121704) and conducted following Preferred Reporting Items for Systematic Reviews and Meta-Analyses (PRISMA) 2020 guidelines, which promote transparent reporting and methodological rigour in evidence synthesis [14]. The primary objective was to evaluate the effectiveness of mHealth applications in reducing alcohol consumption among adults in developed countries. The review process was designed to be comprehensive, reproducible, and minimise bias.

Inclusion and exclusion criteria were structured in Table 1 using the population, intervention, and outcome (PIO) framework. The PIO framework was selected over the more traditional population, intervention, comparison, and outcome (PICO) because the primary objective of this review was to evaluate the effectiveness of mHealth applications for alcohol reduction, rather than to compare them directly against other intervention modalities. Studies were eligible if they included adults aged 18 or older residing in developed countries, implemented mobile phone-based interventions targeting alcohol use or related behaviours, and quantitatively measured changes in alcohol consumption. Exclusion criteria eliminated studies focused on children, conducted in low-income settings, or using alternative intervention modalities such as pharmacological therapy, face-to-face counselling, or web-based tools. Only randomised controlled trials (RCTs) published in English since 2018 were considered, with a focus on populations at highest risk for alcohol-related morbidity.

**Table 1:** Defining inclusion and exclusion criteria using PIO framework.

PIO framework	Inclusion	Exclusion
Population (P)	Adults above 18 years old with alcohol use disorder (AUD) in developed countries.	Children and teenagers with or without alcohol use disorder. Studies that are from developing countries.
Intervention (I)	Mobile phone applications (mHealth).	Conventional intervention, such as pharmacological therapy, face-to-face counselling therapy, group therapy or other types of digital interventions such as internet-based intervention or e-health or telehealth/telemedicine.
Outcome (O)	Reported outcomes on individuals include reductions in alcohol consumption, heavy drinking days (HDDs), risky drinking and alcohol-related behaviours.	Outcome that does not measure alcohol intake.
Study design	Quantitative primary studies, specifically randomised-controlled trials (RCTs) in English language since 2018. Gray literature from top five pages of Ethos.	Qualitative studies and quantitative observational studies in other languages published before 2018.

A comprehensive database search was performed on 1st January 2025 across Cochrane CENTRAL, PubMed, and PsycINFO up to December 2024. Boolean operators and MeSH terms were applied to identify relevant articles. The search steps are demonstrated in Table 2. Keywords included “mobile applications,” “alcohol use,” “adults,” and “developed countries.” Filters were applied to restrict results by language, publication date, and study type. Additional references were identified through manual searching of citation lists [15]. Duplicate records were removed using EndNote X9, streamlining reference organisation.

**Table 2:** The search steps.

Search number	Search terms
Search 1 (P)	“adults” OR “adult” OR “aged” OR “elderly” OR “middle-aged” OR “young adults” OR “older person”
Search 2 (P)	“developed countries” OR “developed nations” OR “first world” OR “high income countries”
Search 3 (I)	“mobile applications” OR “mobile apps” OR “smartphone applications” OR “smartphone app”
Search 4 (O)	“alcohol use” OR “alcohol consumption” OR “heavy drinking” OR “drinking”
Search 5	Search 1 AND Search 2 AND Search 3 AND Search 4

Titles and abstracts were screened independently by two reviewers against predefined eligibility criteria, with full-text review conducted for potentially relevant articles. Where needed, authors were contacted to retrieve missing data. Any discrepancies or uncertainties during screening or data extraction were resolved through discussion and consensus between the reviewers. Quality assessment of included studies was performed collaboratively by both reviewers using the Cochrane risk of bias 2.0 (RoB2) tool [16], which evaluates trials across domains such as randomisation, deviations from interventions, missing data, outcome measurement, and reporting bias. This ensures methodological rigour.

Key data were extracted using structured spreadsheets, including study design, country, intervention type, theoretical framework, sample size, duration, and outcome measures. Given the heterogeneity of interventions and outcomes, a narrative synthesis was conducted to summarise and contextualise findings. Emphasis was placed on clarity and transparency in reporting to support public health applicability. 

Results

**Figure 1:**
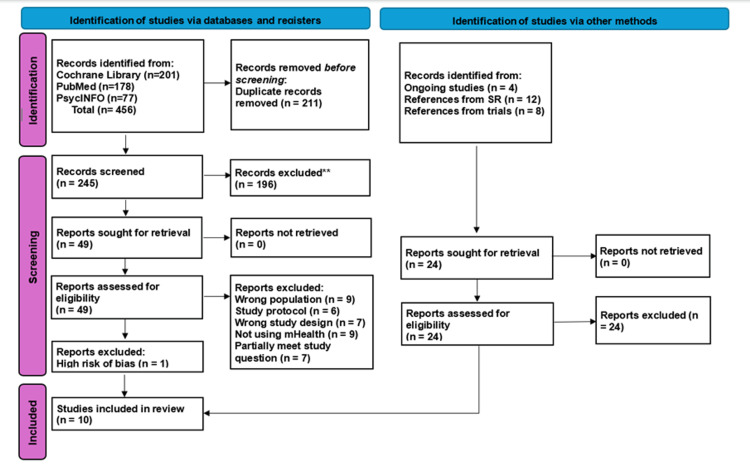
PRISMA flow diagram of study selection process. PRISMA: Preferred Reporting Items for Systematic Reviews and Meta-Analyses.

A total of 456 articles were identified from the Cochrane Library, PubMed, and PsycINFO databases, with an additional 20 found through hand-searching reference lists. After removing duplicates and screening titles and abstracts, 49 full-text articles were assessed. Of these, 38 were excluded based on criteria such as incorrect population, study design, or lack of mHealth interventions. 11 studies were appraised using the Cochrane risk of bias 2.0 tool (RoB 2) [16], with 10 randomised controlled trials (RCTs) included in the final synthesis. This process is detailed in the PRISMA flow diagram (Figure 1).

**Figure 2:**
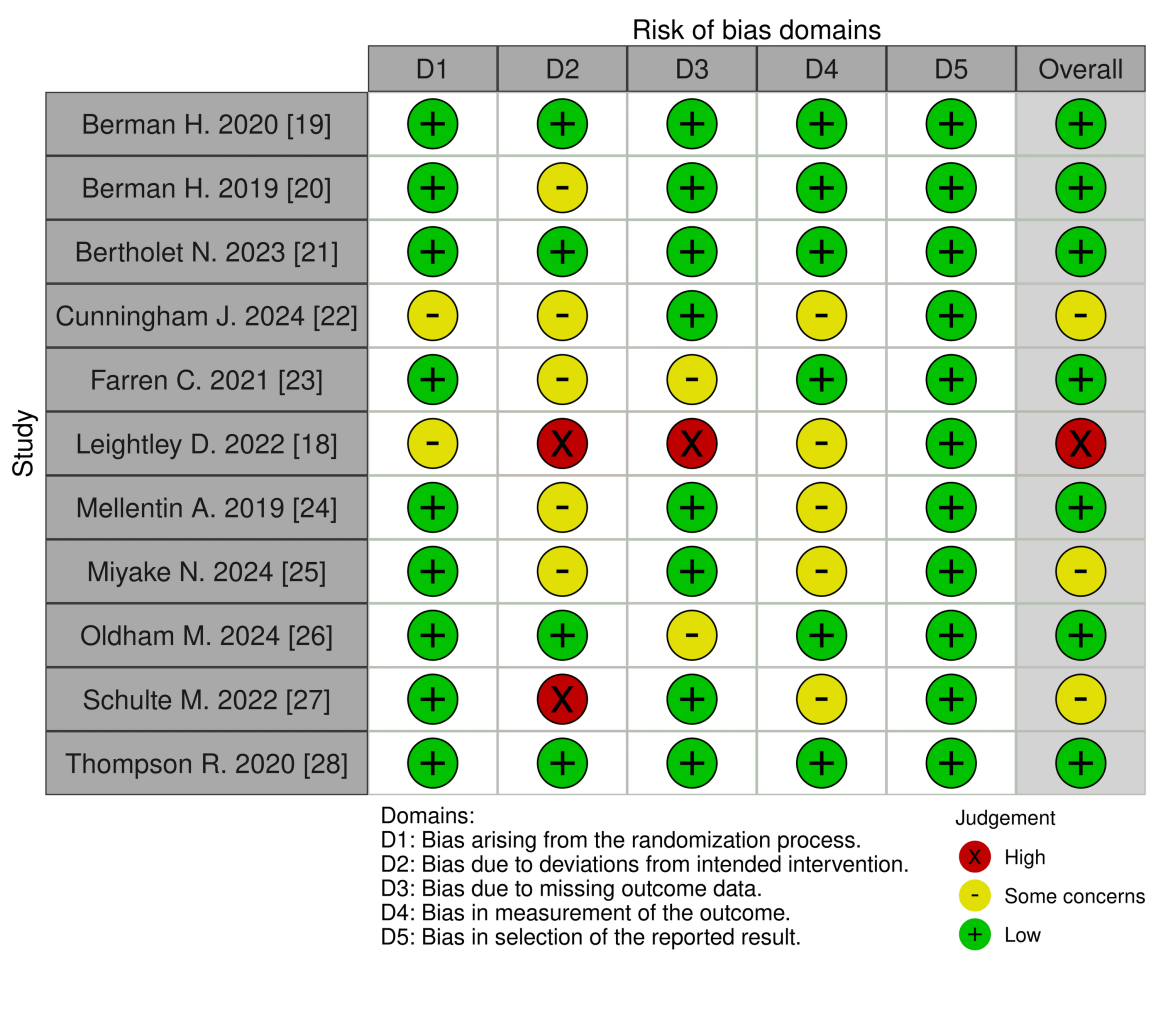
Cochrane RoB 2 risk of bias assessment across included studies. RoB 2: risk of bias 2.0.

Risk of bias assessment was performed across five domains using RoB 2 [17]. Of the 11 appraised studies, Leightley et al. [18] was excluded due to high risk across multiple domains. The domain-level assessments are summarised in Figure 2, which presents a traffic-light style visual of bias across included studies.

The 10 included RCTs involved 11,269 participants across eight developed countries, including Sweden, Switzerland, Canada, Ireland, the UK, Denmark, Japan, and the US. Participants ranged from university students to individuals recovering from alcohol dependence and homeless youth. Ages varied from 19.2 to 53.5 years, with gender distributions between 2% and 87.7% female. A comprehensive breakdown of participant data and study design is summarised in Table 3.

**Table 3:** Characteristics of included studies. IG: intervention group, CG: control group, TAU: treatment-as-usual, CET: cue exposure therapy.

Author(s)/year of publication	Country of origin	Sample size	Context (participants characteristics; previous hazard alcohol consumption (HAC))	Study arm	Funding
Berman et al. (2020) [19]	Sweden	89	Female: 69.7%, n=62; Male: 30.3%, n=27; mean age: 48.93. 39.3% had previous HAC	Two-armed parallel trial first arm (Intervention Group (IG)): TeleCoach intervention app (n=42); second arm (Control Group (CG)): Web-based control app (n=47)	Swedish Alcohol Monopoly Research Council
Berman et al. (2019) [20]	Sweden	2166	Female: 67.5%, n=1462; Male: 32.5%, n=704; mean age: 25.8. University students with previous HAC	Three-armed parallel trial first arm (IG): Promillekoll App (n=722); second arm (IG): PartyPlanner App (n=722); third arm (CG): assessment only control group (n=722)	National Alcohol Monopoly Research Council (Independent). Additional funding: Swedish Research Council
Bertholet et al. (2023) [21]	Switzerland	1770	Female: 54.1%, n=958; Male: 45.9%, n=812; mean age: 22.4. 53.9% had Alcohol Use Disorders Identification Test (AUDIT) scores indicating AUD	Two-armed parallel trial first arm (IG): Smartphone app (n=884); second arm (CG): no access to smartphone app (n=886)	Swiss National Science Foundation
Cunningham et al. (2024) [22]	Canada	761	Female: 56.8%, n=433; Male: 43.2%, n=328; Mean age: 42.0. Participants in the study had previous alcohol problems	Two-armed parallel trial first arm (IG): Full featured app (n=377); second arm (CG): educational only module app (n=384)	Canada Research Chair in Addictions; National Institute of Health and Care Research (NIHR)
Farren et al. (2021) [23]	Ireland	111	Female: 2%, n=111; Male: 98%, n=109; Mean age: 44.3. All participants in this study had AUD, recruited following the completion of a 30-day rehabilitation program	Two-armed parallel trial first arm (IG): Smartphone app “UControlDrink” (UCD) + TAU (n=54); second arm (CG): TAU only (n=57)	Health Research Board of Ireland
Mellentin et al. (2019) [24]	Denmark	164	Female: 22.6%, n=37; Male: 77.4%, n=127; Mean age: 46.0. All participants had AUD, recruited from outpatient alcohol treatment clinic	Three-armed parallel trial first arm (IG): Cue Exposure Therapy (CET) as group aftercare (n=54); second arm(IG): CET as fully mobile phone app aftercare (n=54); thirdarm (CG):Aftercare as usual (n=56)	Lundbeck Foundation, Tryg Foundation
Miyake et al. (2024) [25]	Japan	43	Female: 37%, n=16; Male: 63%, n=27; Mean age: 53.5. All participants diagnosed with alcohol dependence	Two-armed parallel trial first arm (IG): ALM-002 app (n=22); second arm (CG): treatment-as-usual control group (n=21)	CureApp, Inc., the company that developed the ALM-002
Oldham et al. (2024) [26]	United Kingdom	5602	Female: 57.25%, n=3207; Male: 42.22%, n=2365; Mean age: 41.64. Participants in the study were increasing-and-higher-risk drinkers	Two-armed parallel trial first arm (IG): Drink Less app (n=2788); second arm (CG): NHS alcohol advice web page (usual digital care) (n=2814)	NIHR
Schulte et al. (2022) [27]	Netherlands	503	Female: 87.7%, n=441; Male: 12.3%, n=62; Mean age (SD): 22.9 (3.39). No information of previous HAC.	Two-armed parallel trial first arm (IG): Self-guided mobile app “Boozebuster” (n=252); second arm (CG): Educational website on effect of alcohol (n=251)	No funding. However, one of the authors, Anja Huizink received a grant from the European Foundation for Alcohol Research (ERAB)
Thompson et al. (2020) [28]	USA	60	Female: 25%, n=15; Male: 75%, n=45; Mean age: 19.2. Homeless young adults, no information on previous HAC	Two-armed parallel trial first arm (IG): OnTrack (smartphone app) + BMI (n=30); second arm (CG): TAU (n=30)	National Institutes of Health

**Figure 3:**
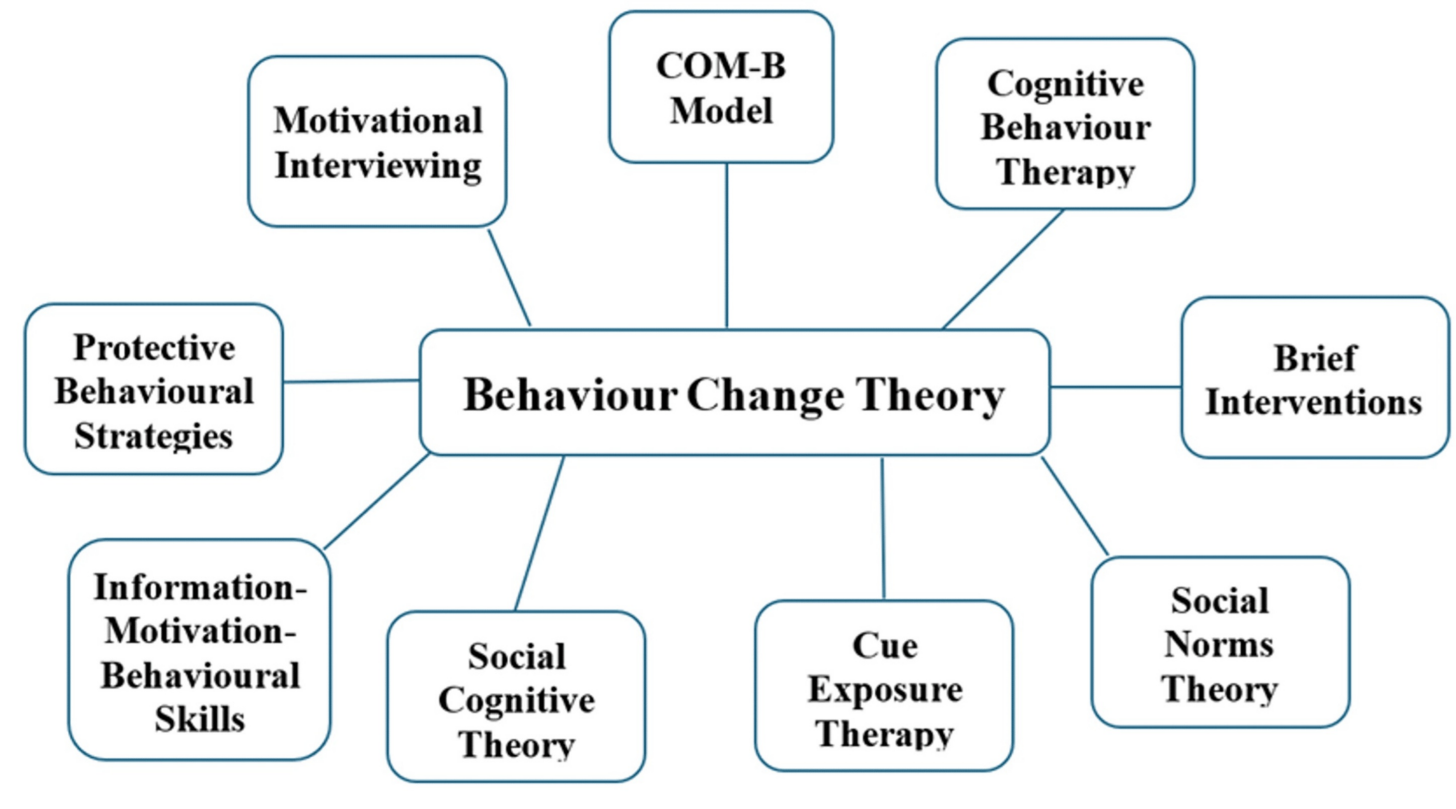
Theoretical frameworks behind app development. COM-B model: capability, opportunity, motivation, and behaviour model.

Behaviour change theory refers to a framework used to understand, predict, and influence human behaviours by identifying the psychological, social, and environmental factors that drive decision-making. The conceptual models guiding app development are illustrated in Figure 3.

Primary outcomes focused on alcohol quantity and frequency, heavy drinking days (HDDs), and standard drinks per week (SD/week), typically measured via timeline follow-back (TLFB) and AUDIT scores. Secondary outcomes included binge drinking, cravings, and alcohol-related consequences. Significant reductions in alcohol use were reported by Berman et al. [20] and Bertholet et al. [21], while Miyake et al. [25] found reductions in HDDs with limited quality-of-life improvements. Thompson et al. [28] highlighted the feasibility but noted a modest behavioural impact.

Apps featured content such as general and personalised education about drinking risks, goal setting, tracking tools, reminders, and motivational prompts. Features like eBAC monitoring and sober-driver designation [21,22] added public safety value. Each intervention was underpinned by behaviour change theory (BCT) and the information-motivation-behavioural skills (IMB) framework, as demonstrated in Table 4, which also outlines the core intervention components and comparative study outcomes. 

**Table 4:** Summary of intervention features and outcomes across studies. IG: intervention group, CG: control group, HDD: heavy drinking days, DD: drinking days, AUDIT: Alcohol Use Disorders Identification Test, AQoLs: Alcohol Quality of Life Scale, ADS: Alcohol Dependence Scale, HAC: hazardous alcohol consumption, SD: standard drink, eBAC: estimated blood alcohol content, CBT: cognitive-behaviour therapy, BCT: behaviour change therapy, PBS: protective behavioural strategies, MI: motivational interviewing.

Author(s) year	Study aim	Theoretical framework	Interventions	Duration of the intervention	Outcome measures	Main outcomes
Berman et al. (2020) [19]	To compare the effects of the TeleCoach app and a web-based control app on the primary outcome of the number of standard drinks consumed per week	(1) Bandura’s Social cognitive theory; (2) CBT; (3) Mindfulness technique	TeleCoach provides information about the consequences of risky drinking. Provides skills-training and relaxation exercises	Six weeks	Quantity and frequency (SD/week). eBAC measured using Windmark formula. Comorbidities: Depression/anxiety/drug use. Self-efficacy regarding abstaining from drinking	SD: Significant within-group decreases (P<0.001), no differences in between-groups (P<0.08). Within-group decreases for binge drink and eBAC for both groups. Self-efficacy: CG>IG
Berman et al. (2019) [20]	To evaluate the effectiveness of mobile application (Promillekoll or PartyPlanner app) in drinking frequencies and binge drinking occasions among university students over 20-week period	(1) CBT; (2) PBS	Promillekoll, PartyPlanner, provide personalised feedback of eBAC, TeleCoach provides skills-training to reduce alcohol consumption. All three apps use PBS as strategies like setting limits on drinking, pacing drinks, and avoiding drinking games.	Twenty weeks; follow-up assessment at seven-,14- and 20-weeks after baseline assessment	Quantity (SD/week). Frequency (drinking occasions/week). Number of binge drinking occasions/week. Mean and peak eBAC.	SD (seven-weeks assessment): Promillekoll>CG (P<0.01) PartyPlanner=CG. Binge drink: Promillekoll.
Bertholet et al. (2023) [21]	To evaluate the effectiveness of providing access to a smartphone application for students with unhealthy alcohol use	(1) BI. (2) Social norms theory (Norms perception)	The mobile app includes six modules designed to provide personalized feedback, compute eBAC enable self-monitoring, set goals, designate a sober driver, and present fact sheets on alcohol and health. These modules aim to engage users by offering normative feedback, estimating risks, tracking drinking patterns, setting drinking limits, and providing educational resources in a user-friendly and non-judgmental environment.	Twelve months; follow-up assessment at three-, six- and 12-months after baseline assessment	Quantity (SD/week). Number of heavy drinking days (HDD). Additional outcomes: Maximum number of drinks over the past 30 days.	SD: IG
Cunningham et al. (2024) [22]	To test the efficacy of a smartphone app targeting unhealthy alcohol consumption in a general population sample.	(1) BI. (2) Social norms theory (Norms perception)	A smartphone, featuring six modules: personalized normative feedback, self-monitoring, goal setting, blood alcohol concentration estimation, designated driver selection, and educational content.	Twenty-three months; follow-up assessment at six months after baseline assessment	Quantity (SD/week). HDD. Number of alcohol-related consequences experienced in the past six months	SD: IG (0.05). Number of alcohol-related consequences: No significant difference between groups (P>0.05)
Farren et al. (2021) [23]	To investigate the treatment response to a six-month intervention using UCD smartphone app	(1) CBT	UControlDrink (UCD); deploying. (1) C-CBT: 12 sessions; (2) Supportive text messages twice daily; (3) Drinking log and recovery log; (4) Personalized craving intervention; (5) Gamification as a reward-based behavioural modification technique.	Six months; follow-up assessment at three- and six months	Number of drinking days (DD), SD per drinking days. HDD AUDIT, cumulative abstinence days (CAD)	DD: No significant difference between groups (P=0.1). SD per drinking days: No significant difference between the groups; HDD: IG
Mellentin et al. (2019) [24]	To examine cue exposure therapy (CET) as group therapy or delivered through mobile phone app is effective as an aftercare.	(1) CET	CET is a behavioural approach to treating AUD by reducing cravings. The app provided adaptive coping strategies and simulated alcohol exposure through videos, allowing users to select their preferred beverage and inducing cravings to practice cue-controlled responses.	Three months followed by aftercare phase which lasted for eight weeks; follow-up assessment immediately after the completion of aftercare phase and six months after completion of aftercare phase.	Quantity (SD/day): Number of DD, HDD. Cravings measured using visual analog scale (VAS). Coping strategies using USCSQ	SD: no significant difference between CET group, IG and CG. Entire sample shows increase DD and HDD at six months after completion of aftercare phase. Cravings: No significant differences were found between IG and CG
Miyake et al. (2024) [25]	To evaluate the efficacy and safety of a therapeutic app (ALM-002) for alcohol dependence in internal medicine clinics. To determine whether the app can reduce heavy drinking days (HDDs).	(1) CBT; (2) BI	ALM-002 analyses the given information and give personalised feedback, triggers and consequences of heavy drinking and recommendations.	Twelve weeks; follow-up assessment at week 24 which is 12 weeks after the end of intervention	HDD: Changes in TAC; ADS AUDIT AQoLs	HDD: significant reduction in IG compared to CG, persisted until week 24. The absolute TAC changes from baseline to week 12: IG (from 86.9 to 41.3; IG from 89.7 to 60.3). AUDIT and AQoLs: No significant between-group differences were observed
Oldham et al. (2024) [26]	To assess the effectiveness the Drink Less app compared to the usual digital care in reducing alcohol consumption.	(1) COM-B	The Drink Less app has eight modules focus on setting goals, tracking progress, planning actions, comparing drinking habits to norms, retraining cognitive biases, gaining insights, substituting behaviours, and providing information about triggers.	Six months; follow-up assessment at three- and six-months after baseline assessment.	Quantity (SD/week); AUDIT score; HDD; alcohol-related consequences	SD: IG
Schulte et al. (2022) [27]	To evaluate the effectiveness of Boozebuster mobile app is in helping young adults reduce their alcohol consumption to stay within low-risk drinking guidelines.	(1) MI; (2) PBS	Boozebuster app includes seven modules including personalized feedback, motivational interviewing, and protective strategies to reduce alcohol consumption.	Six weeks; follow-up assessment at six weeks and three months after the end of intervention	Quantity of alcohol consumption; frequency of alcohol consumption; HDD; alcohol-related problem	Both IG and CG showed similar reductions across all areas, however, there is no significant differences between both IG and CG.
Thompson et al. (2020) [28]	To assess the feasibility and preliminary effectiveness of the OnTrack smartphone application, combined with BMI in reducing substance use and sexual risk behaviours among homeless young adults.	Information-Motivation-Behavioural Skills (IMB) model	OnTrack app helps users track their alcohol and drug use, medication adherence, and sexual risk behaviours daily. It provides personalized feedback, positive reinforcement, and daily tips to build skills, making it an effective and engaging tool for people living with HIV.	Four weeks; follow-up assessment at two- and four-weeks after baseline assessment and two weeks after the end of intervention	Number of drinks; number of times participants drink alcohol before sex	Number of drinks: significant reduction of IG

Behavioural outcomes improved across six studies, independent of participants' previous alcohol use status. Motivation at baseline proved influential: Berman et al. [19,20], Oldham et al. [26], and Schulte et al. [27] found greater reductions among those ready to change. Tailored apps like Promillekoll and Boozebuster reduced binge drinking among youth [20,27], while general population apps like Drink Less showed broader but more gradual effects [26]. However, long-term follow-up was limited; only two studies extended beyond six months.

Across the 10 RCTs, mHealth interventions consistently showed promise in reducing alcohol use, with multifaceted apps integrating feedback, tracking, and motivational strategies yielding the most consistent behavioural changes. The findings support the scalability of these digital tools, especially when tailored to participants' needs and contexts.

Discussion

Effectiveness of mHealth Apps in Reducing Alcohol Consumption

This systematic review evaluated the effectiveness of mobile health (mHealth) applications in reducing alcohol consumption among adults in developed countries. Synthesising data from 10 randomised controlled trials involving 11,269 participants, the findings demonstrate reductions in the quantity and frequency of alcohol use following mobile app interventions. Six studies [19-22,25,28] reported statistically significant outcomes, supporting evidence from Kaner et al. [29] and Magwood et al. [9] that mHealth tools can empower users to manage alcohol intake better.

Mixed Outcomes

Mellentin et al. [24] reported increased drinking days at six months, possibly due to participants' underlying alcohol use disorders requiring more personalised intervention. Although mHealth apps have shown promise across health domains [30,31], clinicians may hesitate to recommend them due to concerns about quality and credibility. Bahadoor et al. [32] warned that some alcohol-related apps may be counterproductive, even promoting drinking. To address this, the UK Royal College of Physicians introduced a Health Informatics checklist to support clinical recommendations [33]. Nevertheless, direct comparisons between mHealth and face-to-face interventions remain limited due to insufficient trials.

Role of Behaviour Change Theories

All reviewed apps were grounded in behaviour change theories (BCTs), though the optimal framework remains unclear due to a lack of standardisation in outcome measures. Studies combining cognitive behavioural therapy (CBT), motivational interviewing (MI), and social cognitive theory showed notable effects [19,20]. Other frameworks included the information-motivation-behavioural skills (IMB) model [28], protective behavioural strategies (PBS), and social norms theory [21,22]. The IMB model’s emphasis on information, motivation, and skills was especially relevant for broad behavioural changes. Apps frequently provided guideline-based advice [21,24], though prior studies found limited impact from information alone [34].

Motivation and Readiness to Change

Motivation appeared crucial: participants who expressed readiness to change, such as those in Berman et al. [19,20], Oldham et al. [26], and Schulte et al. [27], showed greater reductions in alcohol use. This contrasts with Bertholet et al. [21], who found that readiness to change did not predict consumption. External motivators, such as peer stories and goal reinforcement, also played a role.

Impact of Personalised Feedback

Personalised feedback emerged as a particularly impactful feature. Five apps included such feedback, leading to reductions in standard drinks [21,22], binge episodes [20], and heavy drinking days [25]. Petty et al. [35] argued that messages with personal relevance generate lasting behavioural change through deeper cognitive engagement.

Duration, Sample Size, and Measurement Bias

Most studies lasted only four to six weeks, limiting long-term evaluation. Only Bertholet et al. [21] and Cunningham et al. [22] extended beyond 12 months. Longer interventions, incentives, and continuous assessment may enhance outcomes. Small sample sizes in some trials, such as Thompson et al. [28], reduce generalisability. The widespread use of self-reported data further introduces the risk of bias, as participants may underreport or misremember alcohol intake [36]. Objective measures like biomarkers or in-app tracking are recommended for future studies.

Methodological Constraints and Synthesis Approach

The search was limited to three databases, though these are considered comprehensive in the field. Due to variability in BCT frameworks and outcome reporting, meta-analysis was not possible, and a narrative synthesis was deemed the most appropriate analytical approach.

Future Research Directions

Future research should examine which BCTs are most effective and how they are implemented within digital tools. Identifying the “active ingredients” and determining optimal usage patterns or ‘dosage’ could improve intervention design. Many studies lacked data on app engagement, an important predictor of behaviour change given high attrition rates [12]. Research should also address cost-effectiveness compared to traditional care and explore physician and patient perspectives through qualitative methods. Addressing social inequality is vital; as Katikireddi et al. [37] emphasised, alcohol harms disproportionately affect individuals of lower socioeconomic status, and digital interventions could help reduce such disparities.

Public Health Implications and Equity Considerations

These findings highlight the potential of mobile interventions to reduce alcohol consumption at scale. Structured programs incorporating evidence-based BCTs have demonstrated success, particularly among motivated individuals and at-risk groups such as homeless youth [28]. Mobile tools align with current public health guidelines from agencies such as WHO and Public Health England, providing low-cost, accessible interventions that complement traditional services [29]. By integrating digital tools into national strategies and collaborating with app developers, health organisations can expand reach and tailor content to diverse populations. Furthermore, the inclusion of objective measurement tools and standardised endpoints would enhance reliability and promote widespread adoption.

## Conclusions

This review analysed ten recent mHealth studies targeting alcohol reduction, with nine showing positive outcomes. Mobile applications proved effective by offering timely, interactive, and personalised support, especially among motivated users. Compared to traditional methods, mHealth interventions provided modest but meaningful improvements in drinking behaviour and awareness.

Given their scalability and accessibility, mHealth tools have the potential to make a measurable public health impact, particularly in addressing hazardous alcohol use. Future research should focus on optimising core features such as feedback, motivation, and cognitive support to maximise their effectiveness in diverse populations.

## References

[1] Manthey J, Shield KD, Rylett M (2019). Global alcohol exposure between 1990 and 2017 and forecasts until 2030: a modelling study. Lancet.

[2] Shield K, Manthey J, Rylett M (2020). National, regional, and global burdens of disease from 2000 to 2016 attributable to alcohol use: a comparative risk assessment study. Lancet Public Health.

[3] National Health Service (2024 (2024). National Health Service (2024). Health Survey for England, 2022 Part 1: Adult drinking. https://digital.nhs.uk/data-and-information/publications/statistical/health-survey-for-england/2022-part-1/adult-drinking.

[4] Swiss Federal Statistical Office (2024 (2024). Swiss Federal Statistical Office (2024). Swiss Health Survey: Alcohol consumption from 1992 to 2022. https://www.bfs.admin.ch/bfs/en/home/statistics/catalogues-databases.gnpdetail.2024-0167.html.

[5] Sontate KV, Rahim Kamaluddin M, Naina Mohamed I, Mohamed RM, Shaikh MF, Kamal H, Kumar J (2021). Alcohol, aggression, and violence: from public health to neuroscience. Front Psychol.

[6] Saladino V, Mosca O, Petruccelli F, Hoelzlhammer L, Lauriola M, Verrastro V, Cabras C (2021). The vicious cycle: problematic family relations, substance abuse, and crime in adolescence: a narrative review. Front Psychol.

[7] Ryu S (2012). Book review: mHealth: new horizons for health through mobile technologies: based on the findings of the second global survey on eHealth (global observatory for eHealth series). Healthcare Inf Res.

[8] IQVIA (2021 (2024). Digital Health Trends 2021. Trends.

[9] Magwood O, Saad A, Ranger D (2024). Mobile apps to reduce depressive symptoms and alcohol use in youth: a systematic review and meta-analysis. Campbell Syst Rev.

[10] Morris J, Boness CL, Burton R (2023). (Mis) understanding alcohol use disorder: making the case for a public health first approach. Drug Alcohol Depend.

[11] Rimal RN, Real K (2005). How behaviors are influenced by perceived norms: a test of the theory of normative social behavior. Commun Res.

[12] Sawares AS, Shen N, Xue Y, Abi-Jaoude A, Wiljer D (2017). The impact of mobile apps on alcohol use disorder: a systematic review protocol. JMIR Res Protoc.

[13] Hingson RW, Zha W, White AM (2017). Drinking beyond the binge threshold: predictors, consequences, and changes in the US. Am J Prev Med.

[14] Moher D, Liberati A, Tetzlaff J, Altman DG (2009). Preferred reporting items for systematic reviews and meta-analyses: the PRISMA statement. Ann Intern Med.

[15] Horsley T, Dingwall O, Sampson M (2011). Checking reference lists to find additional studies for systematic reviews. Cochrane Database Syst Rev.

[16] Higgins JP, Savović J, Page MJ, Elbers RG, Sterne JA (2019). Assessing risk of bias in a randomized trial. Cochrane Handbook for Systematic Reviews of Interventions.

[17] Sterne JA, Savović J, Page MJ (2019). RoB 2: a revised tool for assessing risk of bias in randomised trials. BMJ.

[18] Leightley D, Williamson C, Rona RJ (2022). Evaluating the efficacy of the drinks: ration mobile app to reduce alcohol consumption in a help-seeking military veteran population: randomized controlled trial. JMIR mHealth uHealth.

[19] Berman AH, Molander O, Tahir M, Törnblom P, Gajecki M, Sinadinovic K, Andersson C (2020). Reducing risky alcohol use via smartphone app skills training among adult internet help-seekers: a randomized pilot trial. Front Psychiatry.

[20] Berman AH, Andersson C, Gajecki M, Rosendahl I, Sinadinovic K, Blankers M (2019). Smartphone apps targeting hazardous drinking patterns among university students show differential subgroup effects over 20 weeks: results from a randomized, controlled trial. J Clin Med.

[21] Bertholet N, Schmutz E, Studer J (2023). Effect of a smartphone intervention as a secondary prevention for use among university students with unhealthy alcohol use: randomised controlled trial. BMJ.

[22] Cunningham JA, Godinho A, Schell C, Studer J, Wardell JD, Garnett C, Bertholet N (2024). Randomized controlled trial of a smartphone app designed to reduce unhealthy alcohol consumption. Internet Interv.

[23] Farren C, Farrell A, Hagerty A, McHugh C (2022). A 6-month randomized trial of a smartphone application, UControlDrink, in aiding recovery in alcohol use disorder. Eur Addict Res.

[24] Mellentin AI, Nielsen B, Nielsen AS, Yu F, Mejldal A, Nielsen DG, Stenager E (2019). A mobile phone app featuring cue exposure therapy as aftercare for alcohol use disorders: an investigator-blinded randomized controlled trial. JMIR mHealth uHealth.

[25] Miyake N, So R, Kariyama K (2024). A smartphone app-based intervention combined with face-to-face sessions for alcohol dependence at internal medicine clinics: a randomized controlled trial. Gen Hosp Psychiatry.

[26] Oldham M, Beard E, Loebenberg G (2024). Effectiveness of a smartphone app (Drink Less) versus usual digital care for reducing alcohol consumption among increasing-and-higher-risk adult drinkers in the UK: a two-arm, parallel-group, double-blind, randomised controlled trial. EClinicalMedicine.

[27] Schulte MH, Boumparis N, Kleiboer A (2022). The effectiveness of a mobile intervention to reduce young adults' alcohol consumption to not exceed low-risk drinking guidelines. Front Digit Health.

[28] Thompson RG, Aivadyan C, Stohl M, Aharonovich E, Hasin DS (2020). Smartphone application plus brief motivational intervention reduces substance use and sexual risk behaviors among homeless young adults: results from a randomized controlled trial. Psychol Addict Behav.

[29] Kaner EF, Beyer FR, Garnett C (2017). Personalised digital interventions for reducing hazardous and harmful alcohol consumption in community-dwelling populations. Cochrane Database Syst Rev.

[30] Dugas M, Gao GG, Agarwal R (2020). Unpacking mHealth interventions: a systematic review of behavior change techniques used in randomized controlled trials assessing mHealth effectiveness. Digit Health.

[31] Wang Y, Min J, Khuri J, Xue H, Xie B, Kaminsky LA, Cheskin LJ (2020). Effectiveness of mobile health interventions on diabetes and obesity treatment and management: systematic review of systematic reviews. JMIR mHealth uHealth.

[32] Bahadoor R, Alexandre JM, Fournet L, Gellé T, Serre F, Auriacombe M (2021). Inventory and analysis of controlled trials of mobile phone applications targeting substance use disorders: a systematic review. Front Psychiatry.

[33] Wyatt JC, Thimbleby H, Rastall P, Hoogewerf J, Wooldridge D, Williams J (2015). What makes a good clinical app? Introducing the RCP Health Informatics Unit checklist. Clin Med (Lond).

[34] Crane D, Garnett C, Michie S, West R, Brown J (2018). A smartphone app to reduce excessive alcohol consumption: identifying the effectiveness of intervention components in a factorial randomised control trial. Sci Rep.

[35] Petty RE, Cacioppo JT, Goldman R (1981). Personal involvement as a determinant of argument-based persuasion. J Pers Soc Psychol.

[36] Anufriyeva V, Pavlova M, Stepurko T, Groot W (2021). The validity and reliability of self-reported satisfaction with healthcare as a measure of quality: a systematic literature review. Int J Qual Health Care.

[37] Katikireddi SV, Whitley E, Lewsey J, Gray L, Leyland AH (2017). Socioeconomic status as an effect modifier of alcohol consumption and harm: analysis of linked cohort data. Lancet Public Health.

